# Remarks upon the Preparation and Modus Operandi of Veratrum Album, or White Hellebore, in the Cure of Inflammatory Rheumatism, Sciatica, Neuralgia, and Croup

**Published:** 1839-01-26

**Authors:** F. W. Adams

**Affiliations:** Maine


*Remarks upon the Preparation and modus operandi of Veratrum Album, or White Hellebore, in the cure of Inflammatory Rheumatism, Sciatica, Neuralgia, and Croup.* By F. W. Adams, M. D., of Maine.

Until the active principle of white hellebore, under the denomination of veratrine or veratria, shall have superseded all other forms of prescription, which I think should not immediately occur, I am inclined to the opinion that none better can be adopted, for internal use, than its vinous tincture, it being convenient for administration, and least likely to produce alarming effects.

It should be prepared by digesting the sliced recent bulbous root, in Spanish white wine, for six or eight days, in a temperature of eighty or ninety degrees, to its fullest saturation. Of this tincture, when filtered, an active dose is from a half to one and a half drachms, according to the age and strength of constitution, from twelve years upwards.

The preparation on which I have mostly, though not exclusively, depended, is three parts of the above, in combination with one part of aromatic vinous tincture, or Sydenham's wine of opium, which I have commonly administered in doses as above, commencing with the least, repeating with the proportional increase of one-half of the preceding dose, every eight hours, until its specific and efficient effects have been procured. These are, a peculiar sensation of warmth, or heat, at the epigastrium, ascending from thence to the fauces, appearing, meanwhile, to penetrate the pulmonary face of the sternum, with fullness and slowness of pulse; which latter symptom goes on increasing until the number of pulsations is reduced, in most cases, to thirty or thirty-five in a minute, and they not unfrequently become extinct at the wrist. The sensation of heat at the sternum, is soon followed by a different one in the fauces, resembling, entirely, that denominated globus hystericus, and accompanied by the same anxiety and sense of suffocation. This, again, is immediately succeeded by nausea and vomiting, which relieve, in a great degree, the foregoing peculiarly distressing sensation. The vomiting, when once excited, commonly continues, almost incessantly, from three-fourths of an hour to an hour and a half, unloading the stomach, effectually, of its alimentary, and, mostly, of its mucous contents. Nearly simultaneous with the commencement of vomiting, a free perspiration occurs, which continues, with a rapid increase, until the patient is literally drenched, the disease seeming to have been totally dissolved and eliminated by cutaneous transpiration; for a state of the most delicious composure succeeds, accompanied by a perfectly quiet and refreshing sleep, which, in a few hours, enables the subject of a long and painful decrepitude, to present himself to his family and friends, in the pleasing contrast of a happy and a healthy man.

I have been in the habit of directing the patient to drink freely of a weak and warm ginger tea, during the continuance of vomiting.

I have administered the above medicine, in cases of croup, which had resisted the effect of almost every popular remedy, to that of full doses of turpeth mineral, and after apparently fatal symptoms had supervened, with the most surprisingly happy result.

I have also prescribed it in several cases of sciatica, or inflammation of the sciatic nerve, with the most complete success. In two cases, a perfect recovery was effected in less than four hours, from a tedious and excruciatingly painful disease. In other cases, where the prescription was embarrassed by the timidity of the patient, as in the cases of several females, the medicine was prescribed in undisturbing doses, and yet, a perseverance in its use was attended with perfectly satisfactory results. The above offers no slight suggestion of its probable efficacy in the whole class of acute neuralgia. I have been induced to try its efficacy, as a diuretic, in several unpromising cases of general dropsy, and in combination with cantharides, with nitric ether, and with digitalis, assisted by external friction and pressure. I have found it to exceed any expectation I had imbibed of its efficacy. I think it a medicine of very active and efficient powers, and one which promises much to the profession, whenever those powers can be judiciously applied. I would remark that I have derived much advantage from its external use, in the form of decoction or infusion, both in local inflammation, and in dropsies, for which I suppose veratria will be more than a substitute.

I have found a decoction or infusion of the root of hellebore, in combination with one drachm of opium to the pint, and used as a lotion, the most effectual application, for the removal of superficial acute local inflammation, of any thing I have seen tried.

Veratria, for all external purposes, may, perhaps, be advantageously substituted for the root.

---

*Fracture of the Clavicle—outer portion the higher.*—A man was brought to the hospital; he had fallen upon his shoulders in the street, and broken his right clavicle, a little beyond the centre. What is curious in the case is, that instead of the humeral fragment being depressed, it is higher than the sternal portion; the fracture is oblique and loose. Put up in the immoveable apparatus.—*Lancet.*

